# Editorial: Atypical Functions of Leukocyte Chemoattractant Receptors

**DOI:** 10.3389/fimmu.2020.596902

**Published:** 2020-10-09

**Authors:** José Luis Rodríguez-Fernández, Mario Mellado, Marcus Thelen, Philip M. Murphy

**Affiliations:** ^1^Department of Molecular Cell Biology, Centro de Investigaciones Biológicas Margarita Salas, Consejo Superior de Investigaciones Científicas, Madrid, Spain; ^2^Department of Immunology and Oncology, Centro Nacional de Biotecnología, Consejo Superior de Investigaciones Científicas, Madrid, Spain; ^3^Faculty of Biomedical Sciences, Institute for Research in Biomedicine, Università della Svizzera Italiana, Bellinzona, Switzerland; ^4^Laboratory of Molecular Immunology, National Institute of Allergy and Infectious Diseases, National Institutes of Health, Bethesda, MD, United States

**Keywords:** chemotaxis, chemokine, atypical chemokine receptor, G protein-coupled receptor, signaling, arrestin, GPCR

Chemoattraction is a process that involves directed cell movement toward extracellular chemical stimuli. It is a fundamental feature of all biological systems that was discovered in the late nineteenth century, when the word “chemotaxis” was coined by the German biologist Wilhelm Pfeffer. Metchnikoff's 1882 description of macrophages from starfish larvae migrating toward an implanted rose thorn irritant represents a seminal observation of chemoattraction *in vivo*. Since then immunologists have described many chemoattractants, including nucleotides, adenosine, lipids (e.g., leukotrienes, prostaglandins, and platelet-activating factor), proteolytic fragments of complement proteins (e.g., C3a and C5a), small peptides (e.g., *N*-formyl peptides), and small proteins (e.g., chemokines, non-chemokine cytokines, and defensins). Chemokines comprise the largest known group of chemoattractants (1, 2).

Despite their structural diversity, chemoattractants typically act by binding to the same class of G-protein-coupled receptors. Chemoattractant system acts appropriately to mediate host defense and tissue repair but can become dysregulated and act inappropriately to mediate immunopathology in the context of autoimmunity and chronic inflammatory disease, including allergic inflammation (3).

The term chemoattractant does not convey the protean activities possessed by many of these molecules that may regulate survival, cytoarchitecture, migratory speed, cell adhesion, reactive oxygen species (ROS) generation, degranulation, endocytosis, differentiation, chemorepulsion, stimulation of immune synapse formation, and release of neutrophil extracellular traps (NETosis), among others (4–6). Chemoattractant multitasking can be viewed as a parsimonious molecular solution to the problem of directing activated immune effector cells to sites of infection and inflammation.

Work on the earliest chemoattractants such as fMet-Leu-Phe and C5a and the first chemokines already showed multiple immunoregulatory activities beyond chemoattraction (7). This was conveyed in the original names that were assigned to some of these chemoattractants, although they were later replaced in the case of the chemokines by a systematic nomenclature (8). For example, neutrophil-activating factor (now CXCL8), macrophage inflammatory protein-1α (now CCL3), and scavenger receptor for phosphatidylserine and oxidized LDL (now CXCL16). This multifunctionality is apparently ancient since it has been observed in unicellular organisms such as *Dictyostelium discoideum* (9). Why then the volume of chemoattractant research has been largest for understanding cell migration?. One explanation is that this is the sole shared property for all of these molecules, which elevates it to a position of general importance. Moreover, cell trafficking is complex with certain general rules, but many exceptions that must be sorted out for each leukocyte subtype, each organ and each tissue barrier.

A fundamental division in the biochemical properties of chemokine receptors has highlighted the division of labor subserved by chemoattractants. The 18 conventional chemokine receptors (cCKRs) drive cell responses by triggering heterotrimeric G protein signaling pathways, whereas a smaller group of atypical chemokine receptors (ACKRs) largely behave as scavengers of chemokines and can use β-arrestins, instead of G proteins, to promote endocytosis and the transport of chemokines to intracellular degradative compartments. The two classes of receptors are oppositely directed and presumably act coordinately to strike a balanced and regulated immune response to prevent a positive feedback loop and runaway inflammation (10).

The study of non-chemotactic functions with modern molecular tools may reveal new insights into how leukocytes transduce simple molecular signals into an integrated and effective immune response in the right place and time. Importantly, chemoattractant receptors may ideally trigger activation as the cells arrive at a focus where the chemoattractant concentration may be highest and above the threshold needed for effector responses. The information gained through the analysis of diverse activities controlled by chemoattractants in leukocytes and the signaling mechanisms involved may be useful to develop strategies to modulate the immune response. The articles of this Research Topic highlight the importance of the non-chemoattractant functions of chemoattractant receptors in cell regulation.

Abouelasrar Salama et al. show that a highly homogeneous rSAA1 [receptor: formyl peptide receptor 2 (FPR2)] induces chemoattraction in leukocytes, synergy with other chemoattractants and monocyte survival. However, they show that commercial SAA1, promotes additional activities in leukocytes caused by contaminants. Their paper represents a note of caution when using commercial ligands to analyze the functions of chemoattractant receptors.

Bianchi and Mezzapelle analyze the role of CXCL12 (receptor: CXCR4) in heart, liver, lung, and peripheral nerve regeneration. They discuss the role of the CXCL12-HMGB1 complex (receptor: CXCR4) in skeletal muscle and bone regeneration. Authors also discuss CXCR4-dependent signaling pathways involved in the regulation of vascular, progenitor, and tumor cell proliferation.

Capucetti et al. analyze the non-chemotactic functions controlled by different chemokine receptors expressed in neutrophils, including CXCR4, CXCR1, CCR1, CCR2, CCR5, and CCR7. The authors analyze the functions regulated by these receptors in different subsets and maturation stages of the neutrophils that are found in multiple physiological and pathological conditions, for example in sites of infection, during autoimmunity, and cancer.

Karin analyses the role of the chemokines CXCL9, CXCL10, and CXCL11 (receptor: CXCR3) and discusses how biased signaling by these ligands affects T cell differentiation and lineage development and may modulate cancer progression and autoimmunity. It is also suggested that CXCL10 and CXCL9 may inhibit cancer cell growth and promote anti-tumor immunity.

Matti et al. focus on the atypical chemokine receptor ACKR4 (ligands: CCL19, CCL20, CCL21, CCL25). The authors show that in the absence of chemokines, β-arrestins interact with ACKR4 and controls the steady-state trafficking of this receptor. Notably, cells lacking β-arrestins can take up CCL19, indicating that these proteins are dispensable for ACKR4-mediated chemokine scavenging.

Morein et al. discuss the effects that the non-conventional activities controlled by chemokine receptors exert on cancer cells. They show that chemokine receptors affect tumor progression by promoting cell proliferation, survival, senescence, enrichment of tumors with cancer stem cells, promotion of metastasis-related functions, such as epithelial-to-mesenchymal transition and increasing the expression of matrix metalloproteinases.

Rodríguez-Fernández and Criado-García analyze the signaling pathways underpinning the different functions controlled by the chemokine receptor CCR7 in dendritic cells. They indicate that CCR7-regulated functions are mediated by highly independent and biased signaling modules. The authors also suggest that other chemoattractant receptors could use a similar strategy to regulate leukocyte functions.

## Author Contributions

All authors listed have made a substantial, direct and intellectual contribution to the work, and approved it for publication.

## Conflict of Interest

The authors declare that the research was conducted in the absence of any commercial or financial relationships that could be construed as a potential conflict of interest.
